# Stroke Experiences and Unmet Needs of Individuals of African Descent Living in High-Income Economy Countries: a Qualitative Meta-Synthesis

**DOI:** 10.1007/s40615-023-01725-z

**Published:** 2023-07-31

**Authors:** Hardeep Singh, Semtetam Patience Fakembe, Racquel K. Brown, Jill I. Cameron, Michelle L. A. Nelson, Kristina M. Kokorelias, Erica Nekolaichuk, Nancy M. Salbach, Sarah Munce, Terence Tang, Carolyn Steele Gray, Arta Taghavi Haghayegh, Heather Colquhoun

**Affiliations:** 1 Department of Occupational Science & Occupational Therapy, Temerty Faculty of Medicine, University of Toronto, 500 University Avenue, Toronto, ON M5G 1V7 Canada; 2 The KITE Research Institute, Toronto Rehabilitation Institute-University Health Network, Toronto, ON Canada; 3 Rehabilitation Sciences Institute, Temerty Faculty of Medicine, University of Toronto, Toronto, ON Canada; 4 Lunenfeld-Tanenbaum Research Institute, Sinai Health, Toronto, ON Canada; 5 Institute of Health Policy, Management and Evaluation, Dalla Lana School of Public Health, University of Toronto, Toronto, ON Canada; 6 Department of Medicine, Geriatrics Division, Sinai Health System, University Health Network, Toronto, ON Canada; 7 Gerstein Science Information Centre, University of Toronto Libraries, University of Toronto, Toronto, ON Canada; 8 Department of Physical Therapy, Temerty Faculty of Medicine, University of Toronto, Toronto, ON Canada; 9 Institute for Better Health, Trillium Health Partners, Mississauga, ON Canada

**Keywords:** Stroke, Meta-synthesis, Perspectives, Culturally tailored, Qualitative, African

## Abstract

**Background:**

Stroke service disparities experienced by individuals of African descent highlight the need to optimize services. While qualitative studies have explored participants’ unique experiences and service needs, a comprehensive synthesis is lacking. To address current knowledge gaps, this review aimed to synthesize existing literature on the experiences of individuals of African descent impacted by a stroke living in high-income economy countries in terms of stroke prevention, management, and care.

**Methods:**

A qualitative meta-synthesis incorporating a meta-study approach was conducted to obtain comprehensive and interpretive insights on the study topic. Four databases were searched to identify qualitative English-language studies published in the year 2022 or earlier on the experiences of adults of African descent who were at risk or impacted by a stroke and living in high-income economy countries. Study methods, theory, and data were analyzed using descriptive and interpretive analyses.

**Results:**

Thirty-seven studies met our inclusion criteria, including 29 journal articles and 8 dissertations. Multiple authors reported recruitment as a key challenge in study conduct. Multiple existing theories and frameworks of health behaviours, beliefs, self-efficacy, race, and family structure informed research positionality, questions, and analysis across studies. Participant experiences were categorized as (1) engagement in stroke prevention activities and responses to stroke symptoms, (2) self-management and self-identity after stroke, and (3) stroke care experiences.

**Conclusions:**

This study synthesizes the experiences and needs of individuals of African descent impacted by stroke. Findings can help tailor stroke interventions across the stroke care continuum, as they suggest the need for intersectional and culturally humble care approaches.

**Supplementary Information:**

The online version contains supplementary material available at 10.1007/s40615-023-01725-z.

## Introduction

Stroke is a high-priority health-related topic of interest in many countries as it is among the leading causes of disability and death [1, 2]. Due to the aging population, the number of people experiencing a stroke in high-income countries, such as the UK, Canada, and European countries, has been growing [2–4]. Moreover, decreases in stroke fatality have resulted in more people living with stroke-related impairments [2], with over half of people who have had a stroke experiencing persistent disabilities/impairments impacting their mobility, vision, speech, and cognition [5]. Psychosocial well-being can also be impacted, as a third of people with stroke experience depression [6]. These stroke-related impacts can reduce long-term functioning and health-related quality of life for people who have had a stroke [6, 7].

For the purposes of this review, ‘high-income countries’ are based on the World Bank’s categorization of countries with high-income economies [8]. Addressing inequities in stroke is a current research and health system priority in many high-income economy countries (e.g. USA, UK) [8–10] due to evidence of ethnically marginalized communities experiencing disparities in the incidence, prevalence, care, and outcomes of stroke [9–13]. Communities such as those of African descent experience a higher risk of a stroke [14], stroke at a younger age [15], and poorer post-stroke functional outcomes (e.g. higher post-stroke morbidity) than White communities [16, 17]. In addition, stroke service disparities, such as unequal treatment and a lower likelihood of referral to stroke treatment, have been previously noted to persist among communities of African descent [18, 19]. There is a clear need for more research to optimize stroke services for communities of African descent [12].

Qualitative research, by virtue of being grounded in a naturalistic paradigm, can provide insight to help better understand lived experiences, including experiences of health and support (e.g. self-management behaviours), and individuals’ needs to maximize their health, health-related quality of life, function and participation, and inform health and social services [20]. Studies such as these have demonstrated that these individuals impacted by a stroke require health providers to consider cultural factors in order to optimize stroke services [21–26]. For instance, a qualitative study with individuals of African descent (eight individuals who have had a stroke, eight primary caregivers, and 18 secondary caregivers) highlighted the importance of considering family members’ needs in stroke interventions as they often play a crucial role in stroke care. Based on their study findings, the author generated a theory to guide considerations for nurses working in acute and community stroke care settings with elderly African American individuals with a stroke and families [21]. Another qualitative study by Pierce highlighted various actions and behaviours grounded in religiosity and cultural beliefs and norms related to caring behaviours, including ‘Christian piety and purpose’ valued by stroke caregivers [23]. The findings of this study were used to create guidance for health providers’ assessment and intervention [23]. While there are some qualitative studies like these that have explored the stroke experiences of communities of African descent impacted by stroke (e.g. [21–25, 27–35]), a comprehensive qualitative meta-synthesis of this literature is lacking [36, 37]. A meta-synthesis of the literature will help inform the development of higher quality and tailored stroke interventions by identifying and addressing relevant contextual factors and barriers and facilitators to implementation. To address the current knowledge gap, the objective of this review was to synthesize existing literature on the experiences of individuals of African descent impacted by a stroke living in high-income economy countries in terms of stroke prevention, management, and care.

## Methods

### Design

We conducted a meta-synthesis incorporating a meta-study approach to identify the similarities and discrepancies reported in findings from multiple sources to produce rich, interpretive insights about this study topic as a ‘coherent whole’ [36–38]. The meta-study approach [39] entails the following steps: (1) formulating a research question, (2) selection and appraisal of primary research, (3) meta-method (examining methods used within), (4) meta-theory (examining theoretical assumptions within studies), (5) meta-data-analysis (coding and reinterpreting findings of studies), (6) meta-synthesis (critically examining theoretical stances within studies and interpreting existing findings), and (7) disseminating findings.

### Formulating a Research Question

The research question we sought to address was: What are the experiences of individuals of African descent impacted by stroke living in high-income economy countries in terms of stroke prevention, management, and care?

*Theoretical approach*: Aligning with a social constructivist paradigm, the interpretations made from the data synthesized from the included studies were co-constructed by the analytic research team members and influenced by their individual experiences and cultures [40–42]. The analytic research team members included HS (occupational therapist, University Assistant Professor who identifies as a woman of South Asian ethnicity residing in Canada), SPF (research assistant with interest in public health who identifies as a woman of African ethnicity residing in Canada), and RB (MSc rehabilitation sciences student who identifies as a woman of Caribbean Chinese ethnicity residing in Canada).

### Selection and Appraisal of Primary Research

*Identifying inclusion/exclusion criteria*: Guided by scoping review methods [43, 44], the inclusion criteria were outlined according to the population, concept, and context (PCC) approach [43] (see Table 1). Studies meeting the following inclusion criteria were included: (i) population: addressed a research question/objective related to the experiences of adults of African descent, (ii) concepts: individuals who were at risk/impacted by a stroke and living in high-income economy countries (i.e. stroke resources/services may differ between higher and lower-income economy contexts) [8, 45], (iii) available in English, (iv) used qualitative methodology to capture participant experiences, and (v) available in full-text and English. No date restrictions were imposed. Dissertations that met the inclusion criteria were included to enhance the comprehensiveness of this review. However, language limits (English language) were applied due to time and resource constraints. Mixed methods studies that did not separately report qualitative data and/or separate findings from participants of African descent were excluded.

**Table 1:** Inclusion criteria

Domain	Inclusion criteria
Population	Individuals of African descent: Adults (≥ 18 years of age) who are of African or African-Caribbean descent who are at risk or have experienced a stroke (i.e. caregiver or person who experienced a stroke). If the study includes other ethnicities, the study should separately report findings from participants of African descent
Concepts	Stroke experiences: The experience of stroke prevention, management, and/or care
Context	High-economy income country, as defined by the World Bank [8]: Andorra, Antigua and Barbuda, Aruba, Australia, Bahamas, Bahrain, Barbados, Belgium, Bermuda, British Virgin Islands, Brunei Darussalam, Canada, Cayman Islands, Channel Islands, Chile, Croatia, Curacao, Cyprus, Czechia, Denmark, Estonia, Faroe Islands, Finland, France, French Polynesia, Germany, Gibraltar, Greece, Greenland, Guam, Hong Kong SAR, China, Hungary, Iceland, Ireland, Isle of Man, Israel, Italy, Japan, Korea Rep, Kuwait, Latvia, Liechtenstein, Lithuania, Luxembourg, Macaro Sar China, Malta, Monaco, Nauru, Netherlands, New Caledonia, New Zealand, Northern Mariana Islands, Norway, Oman, Panama, Poland, Portugal, Puerto Rico, Qatar, Romania, San Marino, Saudi Arabia, Seychelles, Singapore, Sint Maarten, Slovak Republic, Slovenia, Spain, St. Kitts and Nevis, Sweden, Switzerland, Trinidad and Tobago, Turks and Caicos Islands, United Arab Emirates, UK, USA, Uruguay, Virgin Islands (US.)
Literature type	Published full-length empirical studies, including dissertations
Research design	Qualitative methods (including descriptive surveys) *Mixed methods studies were included if they reported qualitative results separately [46] for communities of African descent
Language	Studies available in English

*Specifying appropriate data sources*: The following databases were searched on June 10, 2022: Ovid Medline Epub Ahead of Print, In-Process & Other Non-Indexed Citations, Ovid MEDLINE Daily, Ovid MEDLINE, Ovid Embase, Ovid APA PsycINFO, and Ebsco CINAHL Plus. HS and EN (Faculty Liaison & Instruction Librarian) used relevant subject headings and text words to locate search concepts related to ‘communities of African descent’, ‘high-income countries’, ‘stroke’, and ‘qualitative research’. The search terms were informed by previous reviews conducted by our research team on similar topics. For example, stroke-related search terms were informed by our prior review of the experiences and needs of individuals of South Asian descent impacted by a stroke living in high-income countries [45]. Another prior review conducted by our research team on community-based culturally tailored chronic disease education programs informed the search terms related to individuals of African descent [47, 48]. Supplementary materials 1 presents the database search strategies.

Duplicate articles were removed using Endnote (a reference management software) and Covidence (a review data management website). We also reviewed the reference lists of 10 randomly selected included studies to identify additional relevant studies that may have been missed during the database search.

*Screening and appraisal procedure*: HS and KMK confirmed their understanding of the inclusion criteria by trialing them on a random sample of 25 studies from the search results to ensure consistency among screeners [49]. After this, HS and KMK conducted two screening stages on Covidence. First, HS and KMK independently reviewed the title and abstract of each study to determine whether it met the inclusion criteria listed in Table 1. Then, in the second screening stage, HS and KMK independently reviewed the full text of the studies deemed eligible during the title and abstract screening stage to verify whether it met the inclusion criteria. Finally, disagreements were resolved by HS after re-reviewing the studies.

*Retrieving data*: A data extraction form created by HS to extract data pertaining to author names, year published, research question(s), sample size and participants, type of source (e.g. full-text article, thesis), country, study methods (e.g. design, methods), recruitment strategies, details of the research team (e.g. race-concurrent interviewer or analyzer), study funding source, research aim(s), key findings, including themes, quotes, and researcher interpretations in the discussion related to the needs and experiences of the target population. The form was tested on two articles by SPF and HS. After testing, the form was refined to enhance clarity and comprehensiveness, and then SPF extracted data related to the research questions from all studies on Covidence. In addition, a second reviewer (KMK or HS) reviewed the extracted data for accuracy.

### Data Analysis: Meta-Method, Meta-Theory, and Meta-Data Analysis

HS, SPF, and RB independently examined each of the 37 included studies. Studies presenting data from overlapping samples were treated independently [50]. The methods (e.g. recruitment approach, data collection, analysis, researchers’ positionality) and theories (i.e. theories guiding study and theories produced) were extracted from each study and compared across studies. In line with the meta-study approach [39], the Joanna Briggs Institute’s critical appraisal checklist for qualitative research [51] was used to critically appraise the reported study methods of qualitative studies. It is important to note that the use of checklists to assess the true quality of a qualitative paper qualitative research is contested and these checklists may not align with all qualitative research paradigms [52, 53]. The theories informing studies were descriptively reported.

The meta-data analysis consisted of examining the results (i.e. study themes/categories and subthemes/subcategories) of each study. The interpretive analysis was conducted using Microsoft Word and NVivo 12 (a qualitative data management software) by comparing the results across studies using inductive codes, condensing coded data by combining similar themes/categories, and then interpreting relationships between the data across studies (‘How do these findings fit together with findings from another study?’) [54]. For instance, codes such as ‘turning to God through prayers’, ‘medical care last resort’, and ‘advice and decision-making with social networks’ were categorized into a broader-level category called ‘action after the onset of stroke symptoms’. During weekly team meetings, HS, SPF, and RB’s individual interpretation of the data and emerging coherent findings were discussed, and the meanings were co-constructed to produce the meta-synthesis.

## Results

As outlined in the PRISMA Flow Chart (Supplementary materials 2) [55], 3029 studies were retrieved from the database search. After 908 duplicates were removed, 2120 studies underwent title and abstract screening, and 184 moved on to the full-text review stage. Of these, 37 studies met the inclusion criteria.

An overview of the studies included in this review is presented in Table 2. The studies were journal articles (29/37, 78%) and dissertations (8/37, 22%) and published in the USA (29/37, 78%) and the UK (8/37, 22%). Of the 37 studies, 28 (28/37, 76%) exclusively included community members of African descent, while 9 (9/37, 24%) included mixed ethnic communities (i.e. individuals of African descent and other ethnicities). Moreover, some studies included only caregivers (11/37, 30%), only individuals with stroke (9/37, 24%), individuals with stroke and caregivers (8/37, 22%), individuals with and at risk of stroke or general members of the community (7/37, 19%), individuals with stroke and health providers (1/37, 3%), and individuals with stroke, caregivers, and health providers (1/37, 3%).

**Table 2:** Overview of the studies included in this review

First author, year	Country	Type of article	Contextual details	Ethnicity/immigration	Target sex or gender	Target age	Type of participant
Agi, 2016	USA	Dissertation	Resided < 100 miles from San Diego, CA, for ≥ 2 years	West African-born Immigrants (individuals born in Nigeria (*n* = 4) and Ghana (*n* = 4))	Males	> 18	People at risk/without stroke (*n* = 8)
Anderson, 2017	USA	Dissertation	Prince Georges County, MD	African American members of Christian faith-based organizations	Women	30–65	People at risk/without stroke (*n* = 33)
Bakas et al., 2002	USA	Journal article	Recruited from hospital in large Midwestern City (outpatient neurological clinic)	African American (*n* = 8) and White (*n* = 6)	Female	Unclear	Caregiver (*n* = 14)
Balakrishnan et al., 2017	USA	Journal article	Recruited participants from larger study	Black/African American (*n* = 11) + other ethnicities (*n* = 6)	Not targeted	> 40	Individuals with stroke (*n* = 17)
Beal, 2010	USA	Dissertation	Resided in Texas in private residence or extended care/rehabilitation facility	Not targeted but included: African American (*n* = 1) and others (e.g. Caucasian, Hispanic, African American, Native American and Caucasian ancestry; *n* = 8)	Women	> 21	Individuals with stroke (*n* = 9)
Beal, 2015	USA	Journal article	Recruited from four churches in the USA in two urban areas	African American	Women	> 35	People at risk/without stroke (*n* = 48)
Beal et al., 2012	USA	Journal article	Recruited participants from 2 churches with African American congregants, hair salons, newspapers and in hospitals	Not targeted but included: African -American (*n* = 1) and others (e.g. Caucasian, Hispanic, African American, Native American and Caucasian ancestry; n = 8)	Women	> 21	Individuals with stroke (*n* = 9)
Blixen et al., 2014	USA	Journal article	Recruited from Northeast Ohio, USA	Self-identified as African American	Men with stroke	< 65	Individuals with stroke (*n* = 10) and care partners (*n* = 7)
Blixen et al., 2015	USA	Journal article	Recruited from Northeast Ohio, USA	Self-identified as African American	Men with stroke	< 65	Individuals with stroke (*n* = 10) and care partners (*n* = 7)
Burns et al., 2022	USA	Journal article	Mid-size coastal city in the southeastern USA (stroke belt)	African American (All but one caregiver participant was African American)	Not targeted	> 21 years of age (care partner)	Individuals with stroke (*n* = 20) and caregivers (*n* = 19)
Chang et al., 2018	USA	Journal article	Recruited participants from Los Angeles County	Targeted 4 ethnic communities (self-identified) African American, Chinese American, Korean American, and Latino seniors	Not targeted	> 60	People at risk/without stroke (*n* = 132)
Eaves, 1998	USA	Dissertation	Resided in a rural area, recruited from rural northern counties of North Carolina	African American	Not targeted	> 55	Individuals with stroke (*n* = 8) and primary (*n* = 8) and secondary (*n* = 18) caregivers
Eaves, 2000	USA	Journal article	Resided in a rural area, recruited from rural northern counties of North Carolina	African American	Not targeted	> 55	Individuals with stroke (*n* = 8) and primary (*n* = 8) and secondary (*n* = 18) caregivers
Eaves, 2002	USA	Journal article	Resided in a rural area, recruited from rural northern counties of North Carolina	African American	Not targeted	> 55	Individuals with stroke (*n* = 8) and primary (*n* = 8) and secondary (*n* = 18) caregivers
Eaves, 2006	USA	Journal article	Resided in a rural area, recruited from rural northern counties of North Carolina	African American	Not targeted	> 55	Individuals with stroke (*n* = 8) and primary (*n* = 8) and secondary (*n* = 18) caregivers
Eisenstein et al., 2018	USA	Journal article	Chicago	Targeted African American (*n* = 13), Caucasian (*n* = 9), and Hispanic (*n* = 7) adults	Not targeted	> 18	Individuals with prior stroke, at risk of stroke or had a family/friend with stroke (*n* = 51)
Greene, 2013	USA	Dissertation	One time admission and discharged from inpatient rehabilitation, and resided in South Carolina	African American or Black	Not targeted	> 21	Physical therapists (*n* = 10) and individuals with stroke (*n* = 5)
Greenwood et al., 2014	UK	Journal article	Have used social care (defined as community support by statutory, commercial, or voluntary sector such as personal care, day centre, respite or support group; excludes long-term care or care home; UK)	Self-identified Black African or Black Caribbean (*n* = 10), Asian Indian or Pakistani (*n* = 21), or White British (*n* = 10)	Not targeted	> 45	Carers (*n* = 41)
Greenwood et al., 2016	UK	Journal article	Have used social care (defined as community support by statutory, commercial, or voluntary sector such as personal care, day centre, respite or support group; excludes long-term care or care home; UK)	Self-identified Black African or Black Caribbean (*n* = 10), Asian Indian or Pakistani (*n* = 21), or White British (*n* = 10)	Not targeted	> 45	Carers (*n* = 41)
Greenwood et al., 2017	UK	Journal article	Recently (≤ 2 years) used social care (defined as community support by statutory, commercial or voluntary sector such as personal care, day centre, respite or support group; excludes long-term care or care home; UK)	Self-identified Black African or Black Caribbean (*n* = 10), Asian Indian or Pakistani (*n* = 21), or White British (*n* = 10)	Not targeted	> 45	Carers (*n* = 41)
Johnson, 2014	USA	Dissertation	Residents of Florida recruited from an organization	African American	Not targeted	All ages	Individuals with stroke (*n* = 10)
Jones, 2016	USA	Dissertation	Born and raised in the USA	African American	Men	> 55	Participants with (*n* = 6) and without stroke (*n* = 14)
Lee et al., 2018	USA	Journal article	Recruited through Baptist church site (church members) in an urban area-Washington, DC	African American	Males	> 40	Individuals with stroke and heart disease risk (*n* = 8)
Magwood et al., 2019	USA	Journal article	Recruited from Charleston County, SC area and 3 neighboring counties (Dorchester, Berkeley, and Georgetown)	Individuals with stroke: African American	Not targeted	Unclear	Individual with stroke (*n* = 20), family member/caregiver (*n* = 19) and professional that works with individuals with stroke in health care or community (*n* = 10)
Moorley et al., 2016	UK	Journal article	Lived in the East End of London	African-Caribbean—migrated to England during the Windrush era	Female	Unclear	Individuals with stroke (*n* = 6)
Moorley et al., 2016	UK	Journal article	Now permanently living in East London (UK)	African-Caribbean-born in the Caribbean in St Lucia, Jamaica, or Trinidad	Women	Unclear	Individuals with stroke (*n* = 7)
Moorley et al., 2018	UK	Journal article	Resided in the East End of London (UK)	Self-identify as African-Caribbean	Female	Unclear	Individuals with stroke (n = 6)
Perzynski et al., 2016	USA	Journal article	Participants were from Cleveland, OH	Self-identified African American	Male	< 65	Individuals with stroke (*n* = 10) and care partners (*n* = 7)
Pierce, 1998	USA	Dissertation	From northwestern region of Ohio, resided in same household as care recipient, had verifiable residence	African American (originated from southern United States)	Not targeted	Unclear	Caregivers: primary caregivers/key informants (*n* = 8) and secondary caregivers/general informants (*n* = 16)
Pierce et al., 1999	USA	Journal article	From northwestern region of Ohio; All participants resided in inner-city environments and were satisfied with surroundings/neighbourhood	African American	Not targeted	Unclear	Secondary urban family caregivers (*n* = 16)
Pierce, 2001	USA	Journal article	Urban community in northwestern Ohio	African American	Not targeted	Unclear	Caregivers: primary caregivers/key informants (*n* = 8) and secondary caregivers/general informants (*n* = 16)
Pierce, 2001	USA	Journal article	Urban community in northwestern Ohio	African American	Not targeted	Unclear	Caregivers: primary caregivers/key informants (*n* = 8) and secondary caregivers/general informants (*n* = 16)
Pierce et al., 2001 (coherence)	USA	Journal article	Recruited from rehabilitation setting at a large, urban medical college and hospital; key informants resided in same home as care recipient-urban residence, family income < poverty level, received federal, state or county financial health assistance and did not have private health insurance for individual with stroke	African American	Not targeted	Unclear	Caregivers: primary caregivers/key informants (*n* = 8) and secondary caregivers/general informants (*n* = 16)
Pound et al., 2016	UK	Journal article	Recruited from 11 voluntary sector organizations and local hospital stroke unit in UK	Self-identified Asian Indian (38%), Asian Pakistani (4%), Black African (16%), Black Caribbean (18%) or White British (24%)	Not targeted	> 45	Caregivers (*n* = 50)
Sajatovic et al., 2018	USA	Journal article	US-academic health centre	Self-identified African American	Men	< 65	Individuals with stroke (*n* = 38)
Skolarus et al., 2013	USA	Journal article	Recruited from 3 black churches in Flint, MI; urban community	African American	Not targeted	Adults ≥ 18 and youth (11–16)	Youth (*n* = 39) and adults (*n* = 38) without/at risk of stroke
Strudwick et al., 2010	UK	Journal article	Three urban locations in southern England	Self-identified African-Caribbean ethnicity (i.e. people who originate from the Caribbean Islands or Guyana, and their descendants born in the UK, first and second generation)	Not targeted	Unclear	Carers (*n* = 9)

### Meta-Method

*Publication years*: Studies were published between 1998 and 2022: 1998–1999 (3/37, 8%), 2000–2005 (6/37, 16%), 2006–2010 (3/37, 8%), 2011–2015 (8/37, 22%), 2016–2020 (16/37, 43%), and 2021–2022 (1/37, 3%).

*Author groups*: Several of the studies were published by the same first author, such as Blixen [25, 32], Beal [56–58], Eaves [21, 22, 27, 28], Moorley [33, 35, 59], Pierce [23, 24, 29, 60, 61], and Greenwood [62, 63]. Studies were published in various journals, including stroke or neurology-specific journals (8/37, 22%; e.g. Journal of Stroke & Cerebrovascular Diseases, Annals of Neurology), other health or rehabilitation journals (16/37, 43%; e.g. Journal of Cardiovascular Nursing, Rehabilitation Nursing, American Journal of Health Promotion, Clinical Rehabilitation), or an ethnic or cultural diversity/disparity journal (5/37; 14%; e.g. Ethnicity & Health, Journal of Racial and Ethnic Health Disparities, Journal of Cultural Diversity). The remaining records were dissertations. Studies were funded by agencies such as the American Heart Association, Baylor University, Indiana University School of Nursing, National Institute for Health, University of Michigan, University of Texas, and Veterans Affairs.

*Design*: Most studies employed the following qualitative designs: ethnography [23, 24, 29, 60, 64], community-engaged or community-based approaches [26, 65], grounded theory [21, 22, 27], narrative inquiry [57, 58], or phenomenology [35, 59, 66].

*Recruitment approaches*: Various recruitment strategies were used within studies, including posting advertisements in community programs frequently attended by the target communities, churches, food shops, public libraries, newspapers, and health clinics. In addition, some studies used snowball sampling approaches, including recruitment support from community leaders, health providers, and key informants. Multiple authors noted recruitment challenges with recruiting participants of African descent [21, 27, 67–69], which for some was the most challenging aspect of their study [21, 27]. For instance, one author noted that they could not recruit any participants for nine months [21], thus having to expand their geographical location of recruitment [27]. Another study recommended developing relationships with key informants or visiting culturally tailored social groups rather than study advertisements/posters [68]. Several authors continued to recruit participants until they attained data or theoretical saturation [22, 26–28, 32, 56, 58, 70]. However, other authors indicated that it was difficult to ascertain saturation [21] or that saturation was not applicable to their analysis technique [66]. Instead one preferred content sufficiency, defined as a process of attaining content saturation by creating categories/subcategories using an exhaustive content and systematic data analysis approach [67].

*Data collection*: Studies primarily collected qualitative data using interviews, focus groups, and observations. One author noted a challenge related to group interviews of individuals with stroke and their family members due to family members being less comfortable sharing during these interviews [27]. One study used photovoice methodology [71]. Choice of data collection location appeared to be important as several authors selected sites based on participant comfort and to capture diverse geographical perspectives. For instance, some authors conducted interviews in participants’ home settings [21, 22, 24, 27–29, 33, 35, 59, 60, 64, 67, 68]; one author specified the reason was to enable participants to have a sense of control [22]. Other authors provided participants a choice (e.g. home or community) [58] or selected community locations/activities familiar to them (e.g. doctor’s appointment, dog trainer, community centre, coffee shop, conference room, church) [24, 25, 29, 58, 59, 62, 72]. Authors offered participants refreshments or meals [25, 26, 32, 65, 66, 73]. A photovoice study provided participants with a camera as a gift [71]. Several studies had ethnically concurrent interviewers conduct data collection or involved in data analysis (e.g. [21, 23, 24, 32, 56, 60, 61, 64, 67, 73, 74]).

*Data analysis*: Thematic and content analyses were commonly used as data analysis approaches within studies. In addition, two studies used interpretive phenomenological analysis [35, 59] and four used ethnographic or constant comparative analysis techniques [21, 60, 65, 71].

*Study quality*: Based on Joanna Briggs Institute’s critical appraisal checklist for qualitative research [51], the quality rating of qualitative studies (*n* = 34/37, 92%) ranged between 7 and 10 (average 8.7). Across these studies, they inconsistently reported their philosophical perspectives (*n* = 24/34, 74%), the researchers’ cultural or theoretical perspectives and views (*n* = 16/34, 47%), and the influence of the researcher on the research, and vice versa (*n* = 20/34, 59%).

### Meta-Theory

Authors of some studies used existing theory or framework related to health behaviours, beliefs, self-efficacy, race, and family structure, to inform their research positionality, research questions, and analysis. For instance, authors used the health promotion model [64], health belief model [64, 74, 75], social ecological model of health behaviour [25, 76], symbolic interaction theory [27], Bandura’s theoretical self-efficacy model [65], Bandura’s social cognitive theory [77], critical race theory [67], Common Sense Model of Illness [66, 73], Everet Rogers Diffusion of Innovation Theory [73], and Friedemann’s System Organization Framework [23, 24, 29, 60]. The relationship between culture and stroke experiences were captured as data relating to a community’s values, norms, beliefs (e.g. spirituality) that impact their stroke experiences, while acknowledging that subcultures may lead to differing health beliefs [64, 75]. All authors indicated that understanding stroke experiences from a cultural perspective could improve communication with community members and inform the development of tailored interventions or improve the quality of existing interventions for community members.

### Meta-Data Analysis

*Participants*: Table 2 describes the study samples. Various terms were selected to describe the ethnic identification of the community members, with African American being the most common since studies were primarily conducted in the USA [21–29, 31, 32, 56, 58, 60, 65, 70, 72–78]. However, Chang and colleagues (2018) noted that this term may be problematic as it fails to encompass individuals who are immigrants from African countries [74]. Other terms included Black/African American [67, 71], Black African [62, 63, 69, 79], Black Caribbean [62, 63, 69, 79], African-Caribbean [33, 59, 68], and West African-born immigrants individuals born in Nigeria and Ghana/West African Male immigrants [64]. Due to the ‘vastness of diversity of Africa’, some studies reported focusing on specific communities of African descent. For instance, Agi (2006) focused on immigrants from Ghana and Nigeria [64].

Participants’ (i.e. individuals with stroke and caregivers of African descent) stroke experiences reported across studies were interpreted by the analytic research team and categorized into the following broad categories: (1) engagement in stroke prevention activities and responses to stroke symptoms, including barriers to engaging in stroke prevention activities and responses to stroke symptoms, (2) self-management and self-identity after stroke, and (3) stroke care experiences, including care experiences with health providers and systems and caregiving experiences and expectations.

#### Category 1: Engagement in Stroke Prevention Activities and Responses to Stroke Symptoms

A synthesis of barriers reported across studies to participants’ engagement in stroke prevention (subcategory 1a) and their responses after stroke symptom onset (subcategory 1b) are described in this category.

##### Subcategory 1a: Barriers to Engaging in Stroke Prevention Activities: Health Beliefs and Norms and Structural Barriers

Participant responses from 16 (43%) included studies described various beliefs about stroke prevention held by participants, which impacted participants’ perceptions about the cause of stroke and their health behaviours. Some of these beliefs differed from their healthcare providers, including beliefs related to supernatural causes of stroke which may act as barrier to seeking preventive treatment (e.g. consult a healthcare provider for information about stroke risk reduction, monitoring or treating high blood pressure) or medical treatment for symptoms [59, 64]. Specifically, some participants believed that stroke was a result of a spiritual illness caused by supernatural powers, such as voodoo, juju, witches, and wizards or a punishment from God that required natural or spiritual interventions (e.g. herbs or prayers) instead of medical treatment [59, 64]. While these beliefs were passed down from generation to generation, some participants’ beliefs about supernatural causes of stroke declined over time after immigrating to the USA [64].

Participants in other studies believed that stroke was inevitable due to risk factors, such as hypertension, being so common among African Americans regardless of whether someone engaged in health prevention behaviours (e.g. dietary/lifestyle changes) [56]. The prevalence of hypertension among African American communities appeared to dilute participants’ perceptions of the seriousness of hypertension as a stroke risk factor, as one participant stated: ‘Every Black person that goes to the doctor, they give them the blood pressure pills. So, we don’t take the stroke as seriously because we’re all on blood pressure medicine’ [58]. Overall, several participants, including those with a family history of stroke, had limited knowledge of lifestyle risk factors for stroke [25, 59]. Limited stroke knowledge may also be a result of a lack of readily available information about stroke outside of encounters with health providers, such as public service announcements on the Internet, television, and billboards [56].

Authors reported several reasons participants did not seek preventative medical care. Magwood and colleagues indicated that participants estimated that up to 85% of African American men did not go to the doctor [76]. Participants’ view that ‘Black men were healthier’ than the general public fostered reluctance to access health services [64]. Moreover, other barriers to participants’ engagement in health promotion and stroke prevention activities included having a low perception of their personal stroke susceptibility, despite some having stroke risk factors [25, 26, 56, 57, 59, 66, 75]. In addition, knowledge gaps in participants’ understanding of stroke (e.g. where it occurs in the body) and stroke risk factors (e.g. hypertension) were barriers to accessing preventative health services [22, 25, 56–58, 64, 72, 75, 76, 78]. Additionally, access to health care was limited by structural factors, including accessibility (e.g. geographical distance, transportation difficulties) and affordability of care (e.g. high care costs) [28].

The authors of one study noted that the concept of health prevention was new to immigrants, and they may be influenced by the health-seeking norms within their native countries [64]. For instance, West African-born immigrants were from a culture where the norm was only to visit the hospital when someone was very sick [64]. These individuals were from a culture that rarely sought preventative healthcare or services for mild symptoms because it was ‘like looking for illness to escape family responsibilities’ and ‘associated with lazy men’ [64].

Authors noted that it was common for participants to obtain knowledge regarding stroke from their family members, friends, and acquaintances, while a lesser proportion obtained this information from a health provider [56, 73]. In addition, a lack of publicly available information about stroke and poor communication between participants and health providers was believed to have contributed to participants’ knowledge gaps about stroke prevention [63, 78].

##### Subcategory 1b: Responses to Stroke Symptoms

Participant responses to stroke symptoms were synthesized from 13 studies (35%). While some participants could recognize stroke symptoms and stroke as an emergency requiring a timely response [73], multiple barriers inhibiting timely access to stroke care after the onset of stroke symptoms were described across studies. These barriers included a fear of losing immigration status [65], being burglarized if living in a high-crime area [58, 80], and the possibility of being diagnosed with a medical condition [64]. In addition, past experiences of discrimination from health providers/with care provision [26], medical jargon, being embarrassed or uncertain of their symptoms’ nature and severity, and not viewing the symptoms as an emergency [22, 26, 56, 73] were also barriers to seeking timely medical attention.

After the onset of stroke symptoms, many participants consulted their social network for advice before a health provider [22, 56]. For example, a participant in one of the studies stated, ‘I didn’t go to the doctor for three days. I refused, but they [family] took me anyway’ [73]. Their refusal to seek medical attention was because they ‘did not want to be bothered with doctors…I don’t want to deal with police, firemen, and all that’ [73]. Some caregivers indicated that they avoided seeking medical assistance for the individual with stroke symptoms because they did not want to upset them if they did not view the symptoms as a medical emergency [26, 73].

Instead of seeking immediate medical attention after the onset of acute stroke symptoms, the authors indicated that some participants hid their symptoms [22] or attempted to ‘sleep them off’ [33]. Others relied on prayers to facilitate their recovery [35, 57, 64, 76] or used folk/home remedies [64]. An author reported a quote from participants about why they turned to prayers, ‘we believe that if we have to pray, we cast out the devil that is the cause of the attack. We believe it’s a devilish attack, and if we pray, we cast out the devil that way, this is the belief of Africans’ [64]. As noted above, there were many interacting factors that drove these responses, (including a core belief that a ‘stroke can be prevented by appeasing or destroying the devil’ [64]) which could prevent timely treatment as individuals relied on prayer first instead of seeking timely medical attention [76]. Moorley [35, 59] opined that beliefs like witchcraft could ‘coexist amicably alongside modern medicine’ as these may not necessarily impact medication management or risk factor management. The cost of medical attention was another barrier to seeking health care for stroke symptoms [26, 65] and a motive for turning to God: ‘…people turn to God when in a hopeless situation due to lack of money to seek medical attention…No money, you pray to God to heal you’ [64].

Authors noted that gender roles and expectations grounded in sociocultural beliefs and norms (e.g. the culture of an individual’s native country) impacted participants’ willingness to seek health care [64, 76]. For example, authors noted that men were expected to be strong; being sick and accessing health services (e.g. going to the hospital) contradicted this image, which contributed to men neglecting their health symptoms [25]. Authors noted that participants hiding or ignoring/dismissing stroke symptoms could lead to delayed medical treatment [22, 57]. In contrast, women generally appeared more comfortable seeking health care as they tended to have greater exposure to it (e.g. through childbirth) [64].

#### Category 2: Self-Management and Identity After Stroke

This category includes synthesized data on intra- and inter-personal factors impacting life after stroke, including self-management (subcategory 2a) and self-identity (subcategory 2b).

##### Subcategory 2a: Self-Management

Self-management behaviours from the perspectives of participants related to medications, psychosocial well-being, faith in God, lifestyle changes, and social networks were synthesized from 20 articles (54%).

*Medication management*: Multiple authors reported participant challenges with accessing and managing medication, especially multiple medications, due to the high cost of medication, low income, and lack of insurance; these barriers led to medication deviation [28, 65, 76, 78]. Due to high medication costs, participants requested generic medications or substituted medications with home remedies [25, 28]. Aside from cost barriers, participants in the studies reported concern or confusion about the purpose or side effects of medications, which reduced medication compliance [32, 70, 74, 76].

“I’m very unhappy about taking 21 pills every day and I don’t really know what I’m taking.” [25].

“Clearly some seem to deviate from medication recommendations as indicated by “not taken since daughter went online and found side effects” or deciding that “meds don’t work” without understanding the impact of the decision” [76].

Participants’ beliefs about the effectiveness of alternative and complementary methods (e.g. herbal supplements) led them to deviate from recommended medication regimes as they used these to replace or supplement pharmaceutical medications and instead of medications, home remedies were preferred by some individuals [32, 76].

*Self-managing psychosocial well-being*: Authors indicated that depression was challenging for many participants, including caregivers [31, 33, 35, 70, 76, 78]. Men with stroke had more difficulty self-managing their psychosocial well-being as they had difficulty recognizing and seeking help for their depression [33, 78]. Expressing emotions was difficult for participants with communication challenges, leading to emotional upsets [70]. In addition, some participants were concerned about the social stigma of being labelled as a ‘stroke victim’ and tended to refrain from social settings [76].

*Faith in God*: Self-management strategies used by participants stemmed from culture, religion, faith, and spirituality, including their strong faith in God [35, 66, 72]. Authors identified that belief in God and spirituality positively impacted participants (e.g. their emotional well-being) and supported their stroke recovery [29, 35, 60, 68, 70, 72, 74, 76]. Some participants believed that ‘God is ultimately in charge of every life event’ [72], which helped them stay optimistic about a more positive future and protected their mental health [35, 60, 68]. However, sometimes participants’ beliefs that God could grant recovery led to unrealistic expectations of complete ‘recovery’ [76], or there was a tension between religious beliefs and medical approaches [35].

*Self-management lifestyle changes*: Participants in the included studies described self-management strategies, including conventional lifestyle changes and home remedies informed by cultural beliefs [25, 28]. However, modifying diet was difficult for some due to the high cost of healthy foods [70] and cultural diets (e.g. Soul food) [78].

*Social networks*: Social and religious networks helped participants feel supported after a stroke [26, 35, 59, 67, 72, 76], and peer support groups were an information source and provided emotional support [77]. For example, churches offered support and comfort for participants [26, 35, 59, 67, 72, 76], but it was challenging for some individuals with stroke to attend church due to physical or functional barriers [68, 76].

##### Subcategory 2b: Self-Identity and Stroke

Self-identity after stroke related to gender/social and family role changes, individual responses and returning to work, were synthesized from 12 studies (35%).

*Gender and social/family role changes*: Participants explained that it was common for men to overlook their needs and focus on their family’s needs due to culturally constructed gender roles [64]. However, after a stroke, some men experienced stroke-related changes that no longer allowed them to perform physical work (e.g. being the handyman) or financially contribute to their family (e.g. the breadwinner). Their activity limitations led to emotional challenges related to feeling weak and their role changes [22, 58, 64]. Men also experienced social isolation from family and friends who avoided seeing them ill [25]. In addition, women also had to negotiate a new self-identity after stroke by getting to know their new functions/limitations [33]. Regardless of gender, participants who required physical assistance emotionally struggled as they valued independence and did not want to be a burden to others [22, 57, 76]. In social settings, some participants especially men indicated that they had to be concerned about being profiled for their race and health conditions [25].

*Individual responses*: Participants in the included studies shared individualized responses after stroke related to adopting a new self-identity, accepting self, and engaging with body cues to support stroke recovery [22]. For instance, some participants reported the need to come to terms with their bodies to regain a sense of control [33, 58], and adopting a positive mindset helped deal with stroke recovery [35]. Others isolated themselves from social networks because they were ashamed of their new illness identity [21].

*Returning to work*: Authors noted that participants expressed a desire to return to work due to financial stressors after stroke [72], such as having lower income after having to retire early and the added burdens of medical and health expenses, even for those that had health insurance some expenses were not covered [28].

#### Category 3: Stroke Care Experiences

This category describes participants’ care experiences with health providers and health care systems (category 3a) and caregiving experiences and expectations (category 3b).

##### Subcategory 3a: Care Experiences with Health Care Providers and Health Care Systems

Care experiences with health providers and systems were synthesized from 12 studies (32%). Not all participants trusted health providers [22, 78]. For example, caregivers indicated that they doubted the accuracy of the stroke diagnosis, whether their loved one was receiving proper care or whether a different treatment would be more effective [22, 28]. In another example, Perzynski et al. (2016) found that some participants believed that pharmaceutical companies incentivized the doctor to recommend medications [78]. Participants in the included studies indicated that service providers did not always understand them, including challenges related to their culture or gender [25, 68]: ‘so for doctors to understand us black males as far as what medication to use, what rules to adopt, I don’t think that they know’ [25]. However, some participants did not prefer culturally tailored programs, and in fact felt insulted after being offered services [68]. ‘This is very insulting, in other words they’re saying to me, listen, I can’t help you, go to your own people… she goes to church but that was the Black side of her, but, she would more find more enjoyment being amongst English people... I didn’t really want her to go into, sort of, like, a West Indian day centre, because she wouldn’t have, she didn’t like it’ [68]. Another barrier to patient-provider trust was the providers’ unwillingness to speak to participants about alternative or complementary treatments to pharmaceutical medication [25].

Poor communication with providers made caregivers feel abandoned and overwhelmed because they did not feel like they could ask staff questions about their loved one’s care [63]. Moreover, some participants’ verbal communication (e.g. speaking loudly) was perceived by health providers as rude [63]. Some participants found that the paperwork and procedures in the hospital (e.g. emergency room) were challenging to navigate, and unfamiliar terminology made navigating services difficult [63]. In addition, ongoing paperwork was another barrier for caregivers accessing health support for the individual with stroke [63].

While some participants had overall positive experiences with care [67], some felt they had experienced poor quality of care. For instance, participants indicated they were not involved in determining goals and treatment plans. In addition, they felt the rehabilitation staff appeared to work harder with White individuals during rehabilitation sessions [67]. When they advocated for needed services, participants believed that the staff perceived them negatively [68]. Another example was provided by participants who had experienced difficulty receiving their initial stroke diagnosis; participants had to make repeated trips to the emergency room but were sent home without answers [72]. Care workers arriving late was perceived as disrespectful to the comfort and dignity of the individual with a stroke, and the arrival of an unfamiliar care worker added to caregiver fatigue [79]. In addition, participants perceived disrespect from providers when they took personal calls while working, rushed personal care routines, and used the same washcloth for a person’s intimate and remaining body parts [79]. Participants also felt that dispatchers of emergency services interrogated and discriminated against them due to their ethnicity and if they lived in a lower-income or high-crime community [26].

Participants avoided institutionalized care as carers were concerned about the quality of institutionalized care [68]. However, at the same time, participants perceived relying on formal/paid support or family members for personal care was humiliating and degrading [60]. Beyond health services, a need for additional social support for low-income groups, such as affordable food and exercise programs/facilities [78] and addressing financial barriers to accessing stroke care and medication management, was identified [26, 58].

##### Subcategory 3b: Caregiving Experiences and Expectations

Caregiving experiences and expectations were synthesized from 13 studies (35%). Social and community supports, such as family members/caregivers, friends, and community (e.g. church), were critical in supporting stroke recovery and coping [26, 35, 59, 67, 72, 76]. Authors noted that supportive family/caregiver facilitated recovery from stroke [76].

The social and historical contexts of African culture were believed to influence the caregivers’ expectations and duty to care for or give back to their family members [21, 24, 29]. This duty to care resulted in some caregivers having feelings of personal satisfaction and fulfillment in being able to ‘give back’ [21, 27, 29], but many struggled to manage their other responsibilities (e.g. their own health) while caring for an individual with a stroke [21, 68, 70], resulting in sacrifices to support their loved ones [60].

There were different types of caregiving roles, with some gender-based trends noted, as men tended to provide financial support, whereas women tended to provide emotional and personal care support [21, 24, 29, 60]. Caregivers’ faith in God helped them cope with the demands of caring for someone with a stroke [24, 29, 68]. In addition, due to multiple family members/friends involved in the care, caregivers had to manage others’ opinions/judgements about the quality of their caregiving [21].

## Discussion

To the best of our knowledge, this review presents the first meta-study to advance our knowledge of the experiences and needs of individuals of African descent impacted by stroke living in high-income countries. Based on our analysis, participant experiences and needs after stroke were organized into three categories: (1) stroke prevention and responses to stroke symptoms, (2) self-management and self-identity after stroke, (3) stroke care experiences. While this meta-synthesis is novel, some of the findings are supported by prior literature, discussed below.

### The Need for Targeted Stroke Education

Prior literature has suggested ethnic disparities exist throughout the stroke care continuum [18]. The current meta-study highlights the need for tailored stroke education along the stroke service trajectory, from identifying and responding to stroke symptoms to community re-engagement. Educating individuals about different beliefs about the causes of stroke is necessary, as some stroke beliefs can decrease engagement in healthy behaviours and encourage health-seeking behaviours [22, 56, 57, 64, 70, 81]. Prior research has indicated that individuals of African descent, particularly individuals who are socially disadvantaged, may delay hospital arrival time following the onset of a stroke and have lower usage of acute stroke treatments [82–84]. This is concerning because timely acute treatment results in better outcomes than delayed treatment [85–87]. The reasons for delayed health-seeking behaviour may be due to lower levels of health literacy about specific stroke knowledge domains, challenges identifying and responding to stroke symptoms, and attitudes and behaviours that reduce willingness to seek health services [83, 88]. Additionally, as depicted in the synthesized studies, cultural beliefs about health can sometimes conflict with medical management, with some individuals choosing to resolve their acute symptoms with herbs or prayers while delaying medical treatment [89]. While interventions to enhance stroke knowledge exist [88], the education must be co-designed and delivered in accessible and trusted locations to reach the public, including hair salons, barbershops, churches, TV, and radio, given the importance of acute stroke treatment [48, 65, 81, 90]. While the feasibility and sustainability of stroke intervention delivery in nontraditional settings (e.g. barber shops) require further exploration, these settings are promising for health interventions to have a wide reach and reach high-risk individuals [90, 91]. For example, a 2023 review of health promotion interventions that emphasized community engagement and lived gender and race-based experiences delivered in barbershops found that these interventions positively impacted participant retention and health-related changes [90]. Other reviews of health interventions delivered in faith-based organizations have also noted improved outcomes [92, 93]. However, challenges, tensions, and barriers, such as social, political, economic, institutional, and cultural issues, must be considered [92, 93].

### Cultural Beliefs and Health Behaviours

Cultural beliefs can also impact long-term self-management of stroke symptoms. In some participants’ worldviews, illnesses, such as a stroke, are perceived as disharmony resulting from demons, evil spirits, and supernatural phenomena [64]. The beliefs can have implications on medication adherence and management. The findings from our meta-study provide insights into specific health experiences, beliefs, and norms that may help or hinder engaging in timely stroke care and long-term management, which can be addressed in future health interventions.

### Patient and Provider Trust

To promote positive stroke care experiences and improve trust between patients and healthcare professionals, health professionals may need to implement strategies to build trust during cross-cultural interactions and recognize how their implicit biases may lead to misdiagnosis or disparities in stroke service, including a lower provision of evidence-based treatments [19, 32, 67, 94]. Studies in this review supported the importance of cultural tailoring of interventions and individualization in stroke interventions. This finding aligns with a previous review of culturally tailored chronic health interventions for individuals of Black/African descent, emphasizing the need for cultural tailoring *and* individualization [48] since not every person prefers culturally tailored interventions [68].

### Cultural Tailoring of Stroke Services

Cultural tailoring of stroke services can be achieved by directly involving participants or community members in service design [25, 59]. For instance, various methods have been proposed for cultural tailoring, including ‘culturally competent care’, ‘culturally appropriate communication’, ‘culturally sensitive stroke education’, and ‘culturally relevant therapies’ which acknowledge an individual’s religious or cultural beliefs [31, 35, 56, 67, 78]. Adopting these relevant approaches [35, 56, 67, 78] and an intersectional lens [95] may help improve the quality of stroke care, aligning it better to the unique needs of target populations.

Adopting an intersectional approach to stroke interventions, which critically considers the complex interaction between various elements (e.g. gender, sex, ethnicity) that interact to share one’s experience in the world [95–97], may help effectively address the individual needs of diverse populations of African descent, as their needs and experiences are shaped by multiple cultural and identity factors, such as gender, religion, immigration status, age, and socioeconomic status [98, 99]. For instance, gender identity can impact health behaviours and healthcare use, leading to gendered health disparities [100, 101]. Previous research has found that men of African descent were less likely to access healthcare, including calling 911 after the onset of stroke symptoms, compared to women or White men [83, 102, 103]. Time since immigration and health-seeking norms and beliefs may also differ between individuals born in and those who have immigrated to the country [64]. Additionally, stroke affects individuals of African descent at a younger age than other ethnic groups [104], highlighting the importance of tailored interventions to address age-specific stroke service needs [105]. Beyond health services, there is a need for additional social support for low-income groups, such as affordable food and exercise programs/facilities [78] and addressing financial barriers to accessing stroke care and medication management [26, 58]. Moreover, group differences among individuals of African descent (e.g. Black/African American, Black Caribbean, Black African, West African born) were noted in this review, such as beliefs about supernatural causes of stroke declined over time after immigrating to the USA [64]. These group differences must be considered within interventions but cannot be considered if adopting a one-size-fits-all approach to stroke care. Thus, it is crucial to consider multiple factors in designing and delivering interventions across the stroke care continuum. Individualization may help reduce stereotypical assumptions about individuals based on their ethnicity by carefully assessing each person, including their cultural preferences and religious beliefs [68, 106].

An intersectional cultural humility approach [107] may help providers identify the unique and individual needs of individuals with stroke. Adopting a cultural humility approach requires service providers to learn from their clients and treat them as individuals rather than as a homogenous group [107] can promote the delivery of person-centred care [108]. Actively listening is a key strategy in cultural humility and can help understand patients and build trust between providers and patients [24, 29, 76, 81]. By assessing a person’s cultural contexts and preferences (e.g. religiosity, spirituality), providers can avoid stereotypical assumptions based on ethnicity and determine which culturally tailored resources (if any) may be helpful for that person [24, 64, 81, 106]. For example, if a person is spiritual or religious, integrating spirituality and religion into health promotion and acknowledging the potential benefits that religion and the church could play in stroke recovery may be helpful [32, 59]. By adopting an approach where providers are learning from patients and understanding each patient’s unique needs, providers can acknowledge and attend to differences between individuals and create an open environment for communication [107], thereby deliver person-centred care [108]. For instance, if an individual has poor compliance with medications, providers need to understand the reasons why and may need to educate patients on the side effects and the risks of medication deviation [32].

### Family-Centred Care

The findings of this review resonate with prior research as they highlight the unmet needs of caregivers, suggesting the need for greater care support [109]. In addition, our review found that multiple family members may be involved in caring, so providers may need to help negotiate the division of caregiving duties and educate caregivers and care recipients on the available options if they can no longer provide care (e.g. institutionalized care) [21]. Caregivers are often underrecognized in the health system, but vital sources of support for individuals with stroke [110]. Research has recommended that health providers assess caregivers’ needs and prepare them for their caregiving roles to reduce caregiver burden [110].

### Research-Related Challenges

The studies included in this review provide valuable insights into the experiences and needs of individuals of African descent after stroke. However, multiple authors reported small sample sizes due to recruitment challenges, despite using multiple recruitment strategies, such as recruiting from multiple locations, partnering with churches, community centres, and clinics, and leveraging personal networks. Recruitment challenges are unsurprising as previous research has consistently noted challenges recruiting ethnic minorities for stroke studies [111–113]. To address these recruitment challenges, in 2022, Cunningham-Erves and colleagues developed guidelines for culturally tailored research recruitment, recommending recruitment through multiple channels, establishing relationships with communities and neighbourhoods, and specifying appropriate incentives [114]. In addition, other scholars have recommended using hospital stroke databases and partnering with churches to identify study participants [115]. Hospital stroke databases may also be useful in future research as we note that limited research has captured the experiences and needs of individuals in the hospital. Furthermore, most studies in this review were conducted in the USA and published by the same author groups, highlighting the need for more research from other contexts.

### Limitations and Strengths

This review has some limitations and strengths. First, there is a risk that our review has not captured all the relevant research on this topic [116], despite developing a comprehensive peer-reviewed search strategy in consultation with a librarian and using multiple databases and having two researchers independently screen the titles, abstracts, and full texts. Second, as noted in other meta-studies [116], it is difficult to determine which studies report unique data versus analyzed the same data, including studies that reanalyze data in another study or a dissertation. We treated all studies as unique to avoid incorrect assumptions, which may have affected our findings. Nevertheless, we reported which studies were from the same author group to provide insights into possible common data sets. Third, we only included studies published in English, which may have limited the comprehensiveness of this review. Finally, although two reviewers were involved in data extraction, there is a possibility of data extraction errors.

This qualitative meta-study intended to comprehensively synthesize what is known on this topic based on the existing literature rather than providing generalizations. We used a comprehensive, peer-reviewed search strategy developed with a Faculty Liaison & Instruction Librarian. In addition, our research team included team members who identified as of African/Caribbean descent that were involved in the interpretation of the findings to capture cultural nuances present in the literature. Finally, we enhanced comprehensiveness and rigour with multiple team members involved in data collection and analysis and providing readers with detailed descriptions of the contexts and participants of the included studies, from which interpretations were drawn [117, 118].

## Conclusion

This meta-study of 37 studies provides valuable insights into the experiences of individuals of African descent after stroke and their caregivers, highlighting the need for culturally sensitive care approaches that consider cultural and individual needs. These review findings can inform the development of future tailored stroke interventions across the stroke care continuum. The methodological and theoretical insights generated from this review can guide future research.

## Supplementary Information

Below is the link to the electronic supplementary material.

Supplementary file1 (DOCX 36 KB)

Supplementary file2 (DOCX 50 KB)

## Data Availability

The data that support the findings of this review are available from relevant databases.
