# Supplementary material for: Stroke Experiences and Unmet Needs of Individuals of African Descent Living in High-Income Economy Countries: a Qualitative Meta-Synthesis
Source: J Racial Ethn Health Disparities. 2023 Jul 31;11(5):2608–26. doi: 10.1007/s40615-023-01725-z (PMC11481687; doi:10.1007/s40615-023-01725-z)
Supplement: Supplementary file 1 — Supplementary file1 (DOCX 36 KB) [file 40615_2023_1725_MOESM1_ESM.docx]

Table of Contents

[Medline 1](#_Toc105766465)

[Embase 3](#_Toc105766466)

[APA PsycINFO 5](#_Toc105766467)

[CINAHL Plus 7](#_Toc105766468)

# Medline

Database(s): **Ovid MEDLINE: Epub Ahead of Print, In-Process & Other Non-Indexed Citations, Ovid MEDLINE® Daily and Ovid MEDLINE®**1946-Present
Search Strategy:

| **#** | **Searches** | **Results** |
| --- | --- | --- |
| 1 | blacks/ or african americans/ | 94770 |
| 2 | Racial Groups/ | 24553 |
| 3 | Ethnicity/ | 69408 |
| 4 | Minority Groups/ | 16586 |
| 5 | "Ethnic and Racial Minorities"/ | 331 |
| 6 | black?.tw,kf. | 162816 |
| 7 | (African adj3 (American* or Andorra* or Antigua* or Aruba* or Australia* or Austria* or Bahamas or Bahamian* or Bahrain* or Barbados or Belgian or Belgium or British or Briton? or Brunei* or Canada or Canadian* or Caribbean* or Chile or Chilean* or Croatia* or Cyprus or Czech* or Denmark* or Danish or Estonia* or European* or Finland or Finnish or France or French or German* or Greece or Greek? or Hong Kong or Hungar* or Iceland* or Ireland or Irish or Israel* or Italy or Italian* or Japan* or Korea* or Kuwait* or Latvia* or Lithuania* or Netherlands or Dutch or Holland or New Zealand* or Norway or Norwegian* or Oman or Poland or Polish or Portugal or Portuguese or Puerto Ric* or Qatar or Saudi? or Singapor* or Slovak* or Slovenia* or Spain or Spanish or Swede? or Swedish or Swiss or Switzerland or Trinidad* or UK or United Kingdom or United States or USA or Uruguay*)).tw,kf. | 66683 |
| 8 | (african adj3 (immigrant* or migrant* or refugee* or expat*)).tw,kf. | 1339 |
| 9 | (afro Caribbean or afrocaribbean*).tw,kf. | 1257 |
| 10 | (Africa? adj3 (descent or ancest* or diaspora)).tw,kf. | 5352 |
| 11 | (people of colo?r or person? of colo?r or BIPOC or BAME or BME).tw,kf. | 4582 |
| 12 | (racial factor* or race factor*).tw,kf. | 365 |
| 13 | (mixed-race or mixed racial or mixed ethnic*).tw,kf. | 1532 |
| 14 | ((ethnic* or racial* or race) adj3 (group* or minorit* or health* or communit*)).tw,kf. | 72761 |
| 15 | (minorit* adj3 (group* or communit* or health* or racial* or race)).tw,kf. | 19395 |
| 16 | or/1-15 | 373212 |
| 17 | "intracranial embolism and thrombosis"/ or intracranial embolism/ or exp intracranial thrombosis/ or exp intracranial hemorrhages/ or stroke/ or brain infarction/ or brain stem infarctions/ or lateral medullary syndrome/ or cerebral infarction/ or cadasil/ or dementia, multi-infarct/ or infarction, anterior cerebral artery/ or infarction, middle cerebral artery/ or infarction, posterior cerebral artery/ or hemorrhagic stroke/ or ischemic stroke/ or embolic stroke/ or thrombotic stroke/ or stroke, lacunar/ | 240918 |
| 18 | cerebrovascular disorders/ or hypoxia-ischemia, brain/ | 54153 |
| 19 | stroke rehabilitation/ | 16192 |
| 20 | (stroke* or poststroke*).tw,kf. | 294866 |
| 21 | ((cerebrovasc* or cerebr* vasc*) adj3 (accident* or disorder* or disease* or event* or incident* or infarct*)).tw,kf. | 48104 |
| 22 | ((cerebral or brain* or intracerebral or subarachnoid or "sub arachnoid" or intracranial or intra cranial or subdural or "sub dural") adj3 (infarct* or haemorrhag* or hemorrhag* or ischemia* or ischaemia* or bleed* or thrombos* or thrombus* or embolism*)).tw,kf. | 141574 |
| 23 | or/17-22 | 506225 |
| 24 | exp qualitative research/ or Nursing Methodology Research/ | 87358 |
| 25 | grounded theory/ | 2453 |
| 26 | focus groups/ | 34361 |
| 27 | (qualitativ* or interpretative* or interpretive* or Delphi or themes or focus group* or fieldwork or field work or heuristic* or hermeneutic* or semiotics or narrative* or cluster sample or action research or observational method or constant comparative or theoretical sample or critical* social*).tw,kf. | 501718 |
| 28 | (ethnograph* or ethnonurs* or ethnological or ethnomethod* or autoethnograph* or auto-ethnograph*).tw,kf. | 13586 |
| 29 | ((document* or script* or transcript* or thematic* or critical or discourse* or content) adj2 analy*).tw,kf. | 161173 |
| 30 | interview*.mp. | 451483 |
| 31 | (grounded* adj4 theor*).tw,kf. | 15592 |
| 32 | phenomenolog*.tw,kf. | 31200 |
| 33 | ((document* or script* or transcript* or thematic* or critical or discourse*) adj2 (analy* or interpret*)).tw,kf. | 122745 |
| 34 | ((experience or experiences) adj7 (adult* or survivor* or patient* or individual* or participant*)).tw,kf. | 178544 |
| 35 | (lived experience* or life experience*).tw,kf. | 15987 |
| 36 | (mixed method* or multi method* or multimethod* or mixedmethod*).tw,kf. | 38255 |
| 37 | psychology.fs. | 1152960 |
| 38 | or/24-37 | 2016526 |
| 39 | 16 and 23 and 38 | 873 |

# Embase

Database(s): **Embase Classic+Embase**1947 to 2022 June 09
Search Strategy:

| **#** | **Searches** | **Results** |
| --- | --- | --- |
| 1 | black person/ or african american/ or african caribbean/ | 132903 |
| 2 | multiracial person/ or colored person/ or "creole (people)"/ | 585 |
| 3 | "ethnic or racial aspects"/ or cultural factor/ or ethnic difference/ or ethnicity/ or race/ or race difference/ | 311738 |
| 4 | minority group/ | 17306 |
| 5 | ancestry group/ | 6551 |
| 6 | black?.tw,kf. | 215348 |
| 7 | (African adj3 (American* or Andorra* or Antigua* or Aruba* or Australia* or Austria* or Bahamas or Bahamian* or Bahrain* or Barbados or Belgian or Belgium or British or Briton? or Brunei* or Canada or Canadian* or Caribbean* or Chile or Chilean* or Croatia* or Cyprus or Czech* or Denmark* or Danish or Estonia* or European* or Finland or Finnish or France or French or German* or Greece or Greek? or Hong Kong or Hungar* or Iceland* or Ireland or Irish or Israel* or Italy or Italian* or Japan* or Korea* or Kuwait* or Latvia* or Lithuania* or Netherlands or Dutch or Holland or New Zealand* or Norway or Norwegian* or Oman or Poland or Polish or Portugal or Portuguese or Puerto Ric* or Qatar or Saudi? or Singapor* or Slovak* or Slovenia* or Spain or Spanish or Swede? or Swedish or Swiss or Switzerland or Trinidad* or UK or United Kingdom or United States or USA or Uruguay*)).tw,kf. | 106904 |
| 8 | (african adj3 (immigrant* or migrant* or refugee* or expat*)).tw,kf. | 1727 |
| 9 | (afro Caribbean or afrocaribbean*).tw,kf. | 2222 |
| 10 | (Africa? adj3 (descent or ancest* or diaspora)).tw,kf. | 8300 |
| 11 | (people of colo?r or person? of colo?r or BIPOC or BAME or BME).tw,kf. | 4417 |
| 12 | (racial factor* or race factor*).tw,kf. | 606 |
| 13 | (mixed-race or mixed racial or mixed ethnic*).tw,kf. | 2406 |
| 14 | ((ethnic* or racial* or race) adj3 (group* or minorit* or health* or communit*)).tw,kf. | 94449 |
| 15 | (minorit* adj3 (group* or communit* or health* or racial* or race)).tw,kf. | 24249 |
| 16 | or/1-15 | 641541 |
| 17 | exp cerebrovascular accident/ or exp occlusive cerebrovascular disease/ or exp brain hemorrhage/ or brain embolism/ or exp brain hemorrhage/ or exp brain infarction/ or exp brain ischemia/ or Wallenberg syndrome/ or cerebral artery disease/ or stroke rehabilitation/ | 620591 |
| 18 | (stroke* or poststroke*).tw,kf. | 477489 |
| 19 | ((cerebrovasc* or cerebr* vasc*) adj3 (accident* or disorder* or disease* or event* or incident* or infract*)).tw,kf. | 76011 |
| 20 | ((cerebral or brain* or intracerebral or subarachnoid or "sub arachnoid" or intracranial or intra cranial or subdural or "sub dural") adj3 (infarct* or haemorrhag* or hemorrhag* or ischemia* or ischaemia* or bleed* or thrombos* or thrombus* or embolism*)).tw,kf. | 210673 |
| 21 | 17 or 18 or 19 or 20 | 853248 |
| 22 | exp qualitative research/ | 101387 |
| 23 | qualitative analysis/ | 73751 |
| 24 | nursing methodology research/ | 14819 |
| 25 | grounded theory/ | 8933 |
| 26 | phenomenology/ | 12426 |
| 27 | exp interview/ | 333037 |
| 28 | field work/ | 2024 |
| 29 | exp observational method/ | 7141 |
| 30 | ethnography/ | 3292 |
| 31 | (qualitativ* or interpretative* or interpretive* or Delphi or themes or focus group* or fieldwork or field work or heuristic* or hermeneutic* or semiotics or narrative* or cluster sample or action research or observational method or constant comparative or theoretical sample or critical* social*).tw,kf. | 619910 |
| 32 | (ethnograph* or ethnonurs* or ethnological or ethnomethod* or autoethnograph* or auto-ethnograph*).tw,kf. | 15114 |
| 33 | ((document* or script* or transcript* or thematic* or critical or discourse* or content) adj2 analy*).tw,kf. | 195203 |
| 34 | interview*.mp. | 583203 |
| 35 | (grounded* adj4 theor*).tw,kf. | 19038 |
| 36 | phenomenolog*.tw,kf. | 35533 |
| 37 | ((document* or script* or transcript* or thematic* or critical or discourse*) adj2 (analy* or interpret*)).tw,kf. | 150761 |
| 38 | ((experience or experiences) adj7 (adult* or survivor* or patient* or individual* or participant*)).tw,kf. | 279570 |
| 39 | (lived experience* or life experience*).tw,kf. | 20744 |
| 40 | (mixed method* or multi method* or multimethod* or mixedmethod*).tw,kf. | 44116 |
| 41 | or/22-40 | 1453660 |
| 42 | 16 and 21 and 41 | 1484 |

# APA PsycINFO

Database(s): **APA PsycInfo**1806 to June Week 1 2022
Search Strategy:

| **#** | **Searches** | **Results** |
| --- | --- | --- |
| 1 | blacks/ or "racial and ethnic groups"/ or minority groups/ | 82588 |
| 2 | black?.tw. | 72929 |
| 3 | (African adj3 (American* or Andorra* or Antigua* or Aruba* or Australia* or Austria* or Bahamas or Bahamian* or Bahrain* or Barbados or Belgian or Belgium or British or Briton? or Brunei* or Canada or Canadian* or Caribbean* or Chile or Chilean* or Croatia* or Cyprus or Czech* or Denmark* or Danish or Estonia* or European* or Finland or Finnish or France or French or German* or Greece or Greek? or Hong Kong or Hungar* or Iceland* or Ireland or Irish or Israel* or Italy or Italian* or Japan* or Korea* or Kuwait* or Latvia* or Lithuania* or Netherlands or Dutch or Holland or New Zealand* or Norway or Norwegian* or Oman or Poland or Polish or Portugal or Portuguese or Puerto Ric* or Qatar or Saudi? or Singapor* or Slovak* or Slovenia* or Spain or Spanish or Swede? or Swedish or Swiss or Switzerland or Trinidad* or UK or United Kingdom or United States or USA or Uruguay*)).tw. | 53839 |
| 4 | (african adj3 (immigrant* or migrant* or refugee* or expat*)).tw. | 1179 |
| 5 | (afro Caribbean or afrocaribbean*).tw. | 428 |
| 6 | (Africa? adj3 (descent or ancest* or diaspora)).tw. | 1321 |
| 7 | (people of colo?r or person? of colo?r or BIPOC or BAME or BME).tw. | 2960 |
| 8 | (racial factor* or race factor*).tw. | 227 |
| 9 | (mixed-race or mixed racial or mixed ethnic*).tw. | 1221 |
| 10 | ((ethnic* or racial* or race) adj3 (group* or minorit* or health* or communit*)).tw. | 46487 |
| 11 | (minorit* adj3 (group* or communit* or health* or racial* or race)).tw. | 19925 |
| 12 | or/1-11 | 169988 |
| 13 | cerebrovascular accidents/ | 23104 |
| 14 | cerebral hemorrhage/ or cerebral ischemia/ | 7001 |
| 15 | embolisms/ | 481 |
| 16 | thromboses/ | 917 |
| 17 | (stroke* or poststroke*).tw. | 38826 |
| 18 | ((cerebrovasc* or cerebr* vasc*) adj3 (accident* or disorder* or disease* or event* or incident* or infract*)).tw. | 5357 |
| 19 | ((cerebral or brain* or intracerebral or subarachnoid or "sub arachnoid" or intracranial or intra cranial or subdural or "sub dural") adj3 (infarct* or haemorrhag* or hemorrhag* or ischemia* or ischaemia* or bleed* or thrombos* or thrombus* or embolism*)).tw. | 11246 |
| 20 | 13 or 14 or 15 or 16 or 17 or 18 or 19 | 50443 |
| 21 | qualitative measures/ | 95 |
| 22 | exp qualitative methods/ or mixed methods research/ or exp observation methods/ or phenomenology/ | 41841 |
| 23 | interviews/ or focus group interview/ | 12048 |
| 24 | ethnography/ or ethnology/ | 12261 |
| 25 | (qualitativ* or interpretative* or interpretive* or Delphi or themes or focus group* or fieldwork or field work or heuristic* or hermeneutic* or semiotics or narrative* or cluster sample or action research or observational method or constant comparative or theoretical sample or critical* social*).tw. | 400820 |
| 26 | (ethnograph* or ethnonurs* or ethnological or ethnomethod* or autoethnograph* or auto-ethnograph*).tw. | 34466 |
| 27 | ((document* or script* or transcript* or thematic* or critical or discourse* or content) adj2 analy*).tw. | 83228 |
| 28 | interview*.mp. | 457166 |
| 29 | (grounded* adj4 theor*).tw. | 22882 |
| 30 | phenomenolog*.tw. | 50031 |
| 31 | ((document* or script* or transcript* or thematic* or critical or discourse*) adj2 (analy* or interpret*)).tw. | 53736 |
| 32 | ((experience or experiences) adj7 (adult* or survivor* or patient* or individual* or participant*)).tw. | 98812 |
| 33 | (lived experience* or life experience*).tw. | 33130 |
| 34 | (mixed method* or multi method* or multimethod* or mixedmethod*).tw. | 37819 |
| 35 | (interview or focus group or qualitative study).md. | 432437 |
| 36 | or/21-35 | 983740 |
| 37 | 12 and 20 and 36 | 248 |

# CINAHL Plus

Top of Form

| **#** | **Query** | **Results** |
| --- | --- | --- |
| S1 | (MH "Black Persons+") | 58,212 |
| S2 | (MH "Ethnic Groups") OR (MH "Minority Groups") | 41,990 |
| S3 | TI ( black OR blacks ) OR AB ( black or Blacks ) | 46,316 |
| S4 | TI ( (African N3 (American* or Andorra* or Antigua* or Aruba* or Australia* or Austria* or Bahamas or Bahamian* or Bahrain* or Barbados or Belgian or Belgium or British or Briton* or Brunei* or Canada or Canadian* or Caribbean* or Chile or Chilean* or Croatia* or Cyprus or Czech* or Denmark* or Danish or Estonia* or European* or Finland or Finnish or France or French or German* or Greece or Greek* or Hong Kong or Hungar* or Iceland* or Ireland or Irish or Israel* or Italy or Italian* or Japan* or Korea* or Kuwait* or Latvia* or Lithuania* or Netherlands or Dutch or Holland or New Zealand* or Norway or Norwegian* or Oman or Poland or Polish or Portugal or Portuguese or Puerto Ric* or Qatar or Saudi* or Singapor* or Slovak* or Slovenia* or Spain or Spanish or Swede* or Swedish or Swiss or Switzerland or Trinidad* or UK or United Kingdom or United States or USA or Uruguay*)) ) OR AB ( (African N3 (American* or Andorra* or Antigua* or Aruba* or Australia* or Austria* or Bahamas or Bahamian* or Bahrain* or Barbados or Belgian or Belgium or British or Briton* or Brunei* or Canada or Canadian* or Caribbean* or Chile or Chilean* or Croatia* or Cyprus or Czech* or Denmark* or Danish or Estonia* or European* or Finland or Finnish or France or French or German* or Greece or Greek* or Hong Kong or Hungar* or Iceland* or Ireland or Irish or Israel* or Italy or Italian* or Japan* or Korea* or Kuwait* or Latvia* or Lithuania* or Netherlands or Dutch or Holland or New Zealand* or Norway or Norwegian* or Oman or Poland or Polish or Portugal or Portuguese or Puerto Ric* or Qatar or Saudi* or Singapor* or Slovak* or Slovenia* or Spain or Spanish or Swede* or Swedish or Swiss or Switzerland or Trinidad* or UK or United Kingdom or United States or USA or Uruguay*)) ) | 35,404 |
| S5 | TI ( (african N3 (immigrant* or migrant* or refugee* or expat*)) ) OR AB ( (african N3 (immigrant* or migrant* or refugee* or expat*)) ) | 822 |
| S6 | TI ( ("afro Caribbean" or afrocaribbean*) ) OR AB ( ("afro Caribbean" or afrocaribbean*) ) | 413 |
| S7 | TI ( (Africa OR African) N3 (descent or ancest* or diaspora)) ) OR AB ( (Africa OR African) N3 (descent or ancest* or diaspora)) ) | 1,319 |
| S8 | TI ( ("people of color" or "people of colour" or "person of color" or "persons of color" or "person of colour" or "persons of colour" or BIPOC or BAME or BME) ) OR AB ( ("people of color" or "people of colour" or "person of color" or "persons of color" or "person of colour" or "persons of colour" or BIPOC or BAME or BME) ) | 1,898 |
| S9 | TI ( ("racial factor*" or "race factor*") ) OR AB ( ("racial factor*" or "race factor*") ) | 59 |
| S10 | TI ( ("mixed-race" or "mixed racial" or "mixed ethnic*") ) OR AB ( ("mixed-race" or "mixed racial" or "mixed ethnic*") ) | 593 |
| S11 | TI ( ((ethnic* or racial* or race) N3 (group* or minorit* or health* or communit*)) ) OR AB ( ((ethnic* or racial* or race) N3 (group* or minorit* or health* or communit*)) ) | 32,836 |
| S12 | TI ( (minorit* N3 (group* or communit* or health* or racial* or race)) ) OR AB ( (minorit* N3 (group* or communit* or health* or racial* or race)) ) | 12,219 |
| S13 | S1 OR S2 OR S3 OR S4 OR S5 OR S6 OR S7 OR S8 OR S9 OR S10 OR S11 OR S12 | 148,188 |
| S14 | (MH "Cerebral Infarction") OR (MH "Stroke") OR (MH "Hemorrhagic Stroke") OR (MH "Ischemic Stroke") OR (MH "Embolic Stroke") OR (MH "Stroke, Lacunar") OR (MH "Intracranial Hemorrhage") OR (MH "Cerebral Hemorrhage") OR (MH "Basal Ganglia Hemorrhage") OR (MH "Subarachnoid Hemorrhage") OR (MH "Hematoma, Epidural") OR (MH "Hematoma, Subdural") OR (MH "Hematoma, Subdural, Acute") OR (MH "Hematoma, Subdural, Chronic") OR (MH "Intracranial Embolism and Thrombosis") OR (MH "Intracranial Embolism") OR (MH "Intracranial Thrombosis") OR (MH "Sinus Thrombosis, Intracranial") OR (MH "Cavernous Sinus Thrombosis") OR (MH "Cerebrovascular Disorders") OR (MH "Cerebral Ischemia") OR (MH "Cerebral Ischemia, Transient") OR (MH "Subclavian Steal Syndrome") OR (MH "Hypoxia-Ischemia, Brain") OR (MH "CADASIL") OR (MH "Dementia, Multi-Infarct") OR (MH "Dementia, Vascular") | 109,360 |
| S15 | (MH "Stroke Patients") | 7,186 |
| S16 | TI ( (stroke* or poststroke*) ) OR AB ( (stroke* or poststroke*) ) | 109,421 |
| S17 | TI ( ((cerebrovasc* or "cerebr* vasc*") N3 (accident* or disorder* or disease* or event* or incident* or infract*)) ) OR AB ( ((cerebrovasc* or "cerebr* vasc*") N3 (accident* or disorder* or disease* or event* or incident* or infract*)) ) | 10,649 |
| S18 | TI ( ((cerebral or brain* or intracerebral or subarachnoid or "sub arachnoid" or intracranial or "intra cranial" or subdural or "sub dural") N3 (infarct* or haemorrhag* or hemorrhag* or ischemia* or ischaemia* or bleed* or thrombos* or thrombus* or embolism*)) ) OR AB ( ((cerebral or brain* or intracerebral or subarachnoid or "sub arachnoid" or intracranial or "intra cranial" or subdural or "sub dural") N3 (infarct* or haemorrhag* or hemorrhag* or ischemia* or ischaemia* or bleed* or thrombos* or thrombus* or embolism*)) ) | 28,853 |
| S19 | S14 OR S15 OR S16 OR S17 OR S18 | 164,075 |
| S20 | (MH "Qualitative Studies+") | 169,727 |
| S21 | (MH "Interviews+") OR (MH "Focus Groups") OR (MH "Delphi Technique") OR (MH "Narratives") OR (MH "Observational Methods+") OR (MH "Grounded Theory") OR (MH "Phenomenology") OR (MH "Multimethod Studies") OR (MH "Phenomenological Research") | 311,613 |
| S22 | TI ( (qualitativ* or interpretative* or interpretive* or Delphi or themes or focus group* or fieldwork or field work or heuristic* or hermeneutic* or semiotics or narrative* or cluster sample or action research or observational method or constant comparative or theoretical sample or critical* social*) ) OR AB ( (qualitativ* or interpretative* or interpretive* or Delphi or themes or focus group* or fieldwork or field work or heuristic* or hermeneutic* or semiotics or narrative* or cluster sample or action research or observational method or constant comparative or theoretical sample or critical* social*) ) | 274,360 |
| S23 | TI ( (ethnograph* or ethnonurs* or ethnological or ethnomethod* or autoethnograph* or auto-ethnograph*) ) OR AB ( (ethnograph* or ethnonurs* or ethnological or ethnomethod* or autoethnograph* or auto-ethnograph*) ) | 11,566 |
| S24 | TI ( ((document* or script* or transcript* or thematic* or critical or discourse* or content) N2 analy*) ) OR AB ( ((document* or script* or transcript* or thematic* or critical or discourse* or content) N2 analy*) ) | 81,522 |
| S25 | TI interview* OR AB interview* | 249,474 |
| S26 | TI grounded N4 theor* OR AB grounded N4 theor* | 14,426 |
| S27 | TI phenomenolog* OR AB phenomenolog* | 19,342 |
| S28 | TI ( ((document* or script* or transcript* or thematic* or critical or discourse*) N2 (analy* or interpret*)) ) OR AB ( ((document* or script* or transcript* or thematic* or critical or discourse*) N2 (analy* or interpret*)) ) | 53,611 |
| S29 | TI ( ((experience or experiences) N7 (adult* or survivor* or patient* or individual* or participant*)) ) OR AB ( ((experience or experiences) N7 (adult* or survivor* or patient* or individual* or participant*)) ) OR TI ((life experience* OR lived experience*)) OR AB ((life experience* OR lived experience*)) | 100,761 |
| S30 | TI ( (mixed method* or multi method* or multimethod* or mixedmethod*) ) OR AB ( (mixed method* or multi method* or multimethod* or mixedmethod*) ) | 25,533 |
| S31 | S20 OR S21 OR S22 OR S23 OR S24 OR S25 OR S26 OR S27 OR S28 OR S29 OR S30 | 633,791 |
| S32 | S13 AND S19 AND S31 | 424 |
|  |  |  |

Bottom of Form

Top of Form

Bottom of Form
