# Supplementary material for: Stroke Experiences and Unmet Needs of Individuals of African Descent Living in High-Income Economy Countries: a Qualitative Meta-Synthesis
Source: J Racial Ethn Health Disparities. 2023 Jul 31;11(5):2608–26. doi: 10.1007/s40615-023-01725-z (PMC11481687; doi:10.1007/s40615-023-01725-z)
Supplement: Supplementary file 2 — Supplementary file2 (DOCX 50 KB) [file 40615_2023_1725_MOESM2_ESM.docx]

**Identification of studies via databases and registers**

Records removed *before screening*:

Duplicate records removed (n = 908)

Records identified from*:

Databases (n = 3029)

Medline: n = 873

Embase: n = 1484

PsychINFO: n = 248

CINAHL Plus: n = 424

**Identification**

Records screened

(n = 184)

Records excluded**

(n = 1937)

Reports sought for retrieval

(n = 184)

Reports not retrieved

(n = 1)

**Screening**

Reports assessed for eligibility

(n = 184)

Reports excluded:

Wrong study design (n = 64)

Wrong condition or age (n = 29)

Did not separately report results for participants of African descent (n = 18)

Wrong setting (n = 10)

Different or unclear ethnicity of participants (n = 19)

Conference abstract (n = 8)

Wrong concept (n = 1)

Studies included in review

(n = 37)

Reports retrieved via hand search

(n = 1)

**Included**

*Consider, if feasible to do so, reporting the number of records identified from each database or register searched (rather than the total number across all databases/registers).

**If automation tools were used, indicate how many records were excluded by a human and how many were excluded by automation tools.

*From:*  Page MJ, McKenzie JE, Bossuyt PM, Boutron I, Hoffmann TC, Mulrow CD, et al. The PRISMA 2020 statement: an updated guideline for reporting systematic reviews. BMJ 2021;372:n71. doi: 10.1136/bmj.n71

For more information, visit: <http://www.prisma-statement.org/>
